# Treatment of Delirium Tremens

**Published:** 1878-05

**Authors:** 


TREATMENT OF DELIRIUM TREMENS.—C. S. Wills states, in the *British Medical Journal*, February 2, that he has used capsicum for more than twelve years in the treatment of delirium tremens, with unvarying success; it has never failed, no matter how violent the patient may have been. In extreme cases, thirty grains in bolus may be given every hour, but milder cases simply require smaller doses.

